# Optimizing the Maastricht Work-Related Support intervention in clinical patient care: the value of integrating action research into intervention mapping

**DOI:** 10.1186/s12913-024-10752-3

**Published:** 2024-03-11

**Authors:** Maarten Butink, Shari Hooper, Annelies Boonen, Vera Baadjou, Tim Boymans, Marieke Pierik, Angelique de Rijk

**Affiliations:** 1 Department of Internal Medicine, Division of Rheumatology, Maastricht University Medical Centre+ (MUMC+), P. Debyelaan 25, Maastricht, 6229 HX The Netherlands; 2 Department of Social Medicine, Care and Public Health Research Institute (CAPHRI), Maastricht University, Duboisdomein 30, Maastricht, 6200 MD The Netherlands; 3 Care and Public Health Research Institute (CAPHRI), Maastricht University, Duboisdomein 30, Maastricht, 6200 MD The Netherlands; 4 Department of Rehabilitation Medicine, Care and Public Health Research Institute (CAPHRI), Maastricht University, Universiteitssingel 40, Maastricht, 6200 MD The Netherlands; 5 Department Orthopedic Surgery, Maastricht University Medical Centre+ (MUMC+), P. Debyelaan 25, Maastricht, 6229 HX The Netherlands; 6 Department of Internal Medicine, Division of Gastroenterology and Hepatology, Maastricht University Medical Centre+ (MUMC+), P. Debyelaan 25, Maastricht, 6229 HX The Netherlands

**Keywords:** Work participation, Intervention, Clinical care, Action research, Qualitative design

## Abstract

**Background:**

Healthcare professionals (HCPs) are increasingly recommended to play an important role in supporting people with chronic disease in work participation. An intervention for HCPs to provide work-related support to their patients in clinical care was developed with intervention mapping (Maastricht Work-Related Support; Maastricht WRS). Action research proposes ‘combining research and practice’, which allows us to incorporate experiences of HCPs while implementing and to realize intervention’s full potential. Therefore, the aim of this study is to explore, by integrating action research into an intervention mapping approach, how experiences of HCPs with early implementation can be used to optimize the Maastricht WRS in clinical care.

**Methods:**

Semi-structured interviews were held with nine HCPs (response rate 82%), involved in care for people with inflammatory arthritis, knee problems or inflammatory bowel disease. Some of them were not yet trained in the Maastricht WRS while others had received the training and were providing the Maastricht WRS.

**Results:**

All participants regarded WRS an important part of clinical care. Untrained HCPs indicated a lack of knowledge and skills in providing the Maastricht WRS, and a need for tools. Trained HCPs were satisfied with the training and tools, but stressed that practical limitations hindered providing the Maastricht WRS. Action research showed that the intervention meets the needs of HCPs, but need some optimizations: (1) organizing ‘intervision’ for HCPs, (2) inform and activate patients to discuss work with their HCP, (3) update initial tools and (4) including patients’ work status in the electronic patient system.

**Conclusions:**

Action research integrated into intervention mapping proved to improve the Maastricht WRS intervention. By involving HCPs, the intervention could be optimized to provide to support people with chronic diseases in clinical care in healthy and sustainable work participation.

**Supplementary Information:**

The online version contains supplementary material available at 10.1186/s12913-024-10752-3.

## Background

**Fig. 1:**
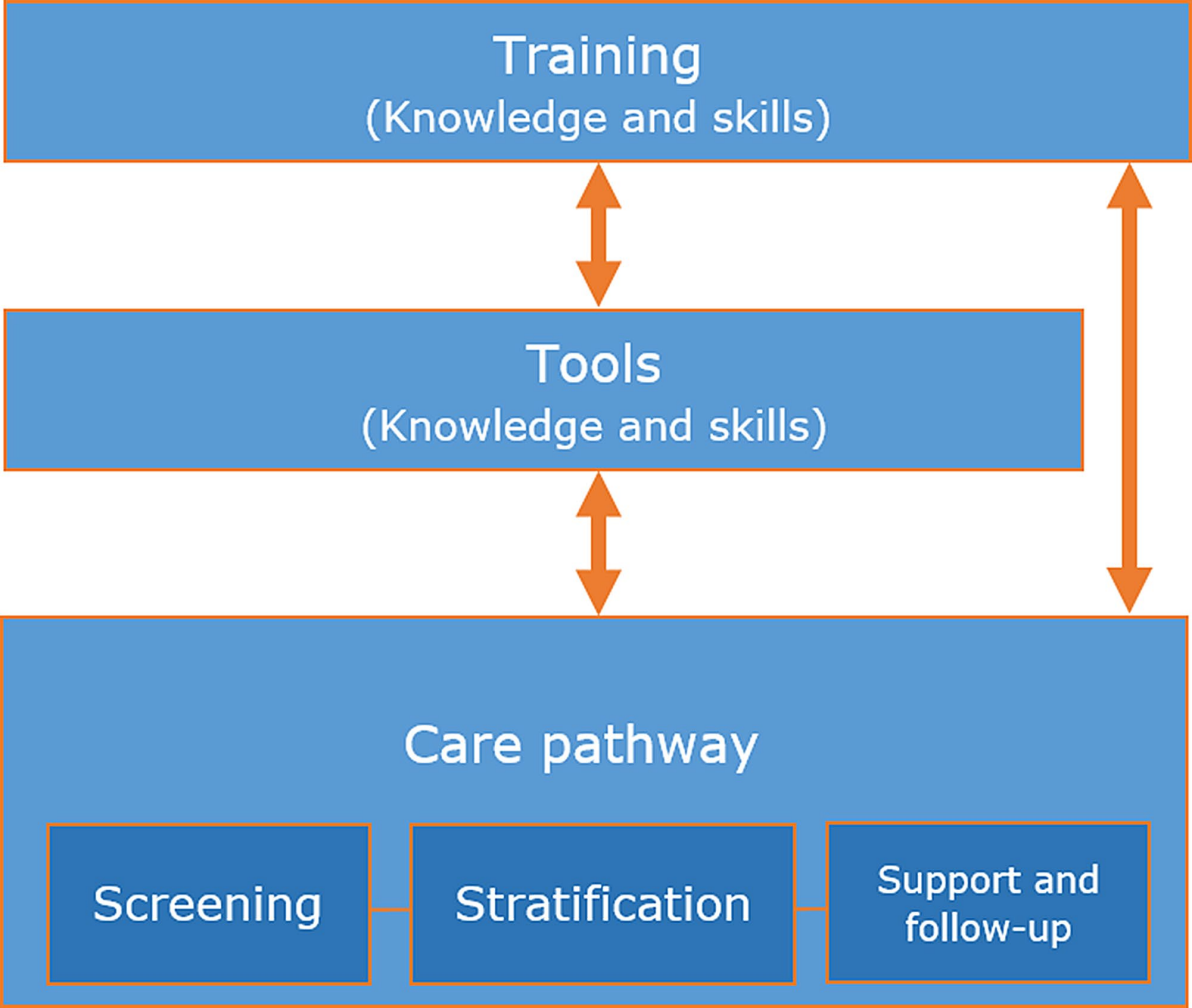
Overview of the components (care pathway, trainings and tools) constituting the Maastricht WRS intervention

The number of people in working age with chronic diseases is increasing worldwide, causing considerable repercussions for work participation [1]. Chronic diseases require complex management over an extended period, involving a variety of healthcare professionals (HCPs), medicinal remedies and monitoring systems [2]. In the Netherlands, over half of the population (9 million) has at least one chronic disease. Employment rates of people with one or more chronic diseases in the Netherlands are lower compared to other wealthy West-European countries [3]. Despite efforts in social legislation to promote work participation, a high number of sick leave prevails [4]. Clinical and occupational care in the Netherlands are traditionally segregated, and this split resulted in neglecting the topic of work participation by HCPs in disease management. Consequently, support to maintain people with chronic diseases at work is often initiated late in the disease trajectory, when opportunities to prevent work loss might be forgone. Moreover, self-employed workers do not have public access to occupational healthcare and only a small minority can afford to insure themselves privately [4]. National socioeconomic and medical advisory reports, and scientific literature endorse the need to identify work-related problems early in the disease trajectory embedded in regular clinical care and suggest to provide work-related support (WRS) [5–8]. Previous interventions on WRS provided in clinical care setting appeared to have limited effects on work outcome. However, these interventions were not truly integrated into usual care, and HCPs were only asked to refer patients who met specific criteria for a particular intervention [9–12]. To this end, a new intervention (Maastricht Work-Related Support; Maastricht WRS) was developed that aimed to integrate, throughout the entire patient’s journey, WRS into regular clinical care by focusing on the patient’s initial HCP. For this development, the first four steps of the intervention mapping (IM) approach were used to co-create the intervention with HCPs, patients, and experts on WRS in clinical care. During development of the Maastricht WRS it appeared important to target the behavioral change of HCPs, by improving their attitude, self-efficacy and social influence. Behavioral change in this intervention is important because HCPs need to integrate the provision of WRS into their daily clinical practice. Since this is not yet common practice, the Maastricht WRS intervention includes three components, including: (1) a care pathway describing how to provide WRS in clinical care, (2) training sessions for HCPs to provide insight into the background and content of the Maastricht WRS, and (3) practical tools to support HCPs performing the different tasks within the Maastricht WRS (Fig. 1). In the care pathway, HCPs need to (a) screen those patients at risk, (b) identify the problem and stratify, (c) provide support themselves and follow-up their patients. In case patients are stratified with high-complexity problems, HCPs can refer patients to a new work participation clinic with expertise in supporting this specific patient group. Three outpatient clinics - caring for people with inflammatory arthritis, inflammatory bowel diseases, and people suspected for knee osteoarthritis - agreed to participate in the intervention; nurse specialists and medical specialists from two of these already participated in the training sessions and started providing the Maastricht WRS in daily practice. A detailed overview of the intervention has been published [13].

In intervention development using IM, needs are initially assessed extensively (Step 1), followed by the development of an intervention (Steps 2–4), and subsequent implementation and evaluation (Steps 5, 6). This cycle leads to the implementation of interventions in practice, with the opportunity to optimize the intervention only after evaluation, given the static nature of IM that facilitates intervention testing only to a limited extent. However, there is no template or structure to test the interventions during the early phase of implementation with the aim of optimizing the intervention. The action research approach could provide a structure to combine experiences from practice and research by exploring HCPs’ experiences and gathering optimizations for the Maastricht WRS, including the training for HCPs. Action research is described as a method to combine research and practice, and guides researchers to better understand daily practice by involving participants along the development of an intervention [14, 15]. Through this approach, early experiences, needs, barriers, and facilitators to provide Maastricht WRS in clinical care might be identified among HCPs, and this input will serve as additional needs to supplement the original needs as derived in Step 1 of IM [13]. With this approach, the intervention’s full potential can be achieved by trying to better match the HCPs’ needs to the intervention, and prevent implementation failure.

Therefore, the aim of this study is to explore, by integrating action research into an intervention mapping approach, how experiences of HCPs with early implementation can be used to optimize the Maastricht WRS intervention in clinical care.

## Methods

### Design

Semi-structured interviews were conducted to explore experiences of HCPs with early implementation [16].

### Participant selection and setting

A purposive sample of HCPs, including medical specialists and nurse specialists, of four outpatient clinics at a Dutch university hospital (Maastricht University Medical Centre^+^) participating in the Maastricht WRS, was invited. At the time of this study, a group of HCPs had already received formal training and provided support, while others were either not trained or had not yet provided support. As we aimed to implement the Maastricht WRS intervention gradually in the hospital, we needed also the experiences of the untrained HCPs at the moment of the early implementation, which was about one year after we started developing the intervention. This approach aimed to gather insights from all intervention users (HCPs) in different stages of behavioral change, to understand their experiences and needs to optimize the total intervention [17]. Numerically more HCPs from one outpatient clinic (rheumatology) were invited, because at this outpatient clinic the intervention started earlier compared to the other clinics. Nine out of eleven contacted HCPs (response rate 82%) agreed to participate. Reasons for non-response were not inquired. Six out of nine HCPs were medical specialists (67%), and three were nurse specialists. HCPs were working at the department of rheumatology (5/9; 56%), orthopedics (2/9; 22%), or gastroenterology (1/9; 11%), and one medical specialist in rehabilitation medicine (1/9; 11%) who was involved in providing complex work-related support. The majority were women (5/9; 56%), and had received training to provide the Maastricht WRS (6/9; 67%).

### Data collection

A semi-structured interview guide was developed based on the interviews conducted for the needs assessment when developing the intervention [13]. The questions aimed to stimulate a discussion around four predefined topics: (1) experience with WRS before training for the Maastricht WRS, (2) the perceived need for providing the Maastricht WRS, (3) experiences or expectations of the training session to gain knowledge and skills on providing work-related support (adapted to whether the participant had received the training or not), and (4) the barriers and facilitators for providing the Maastricht WRS in practice. The interview guide was adapted to HCPs who had already participated in the training or provided the Maastricht WRS (Supplementary Table 1).

Interviews were conducted between April and July 2020. The interviewer (SH) received training in communication techniques before starting the formal interviews. The interviews took place online (video call) or in the hospital, and lasted between 20 and 70 min. The interviewer made notes during all interviews, and all but one were recorded after informed consent. All participants provided informed consent. This study was part of an ongoing study, and was checked for ethical consideration (reference MUMC^+^ 2021–3001).

### Data analysis

Firstly, recorded interviews were anonymized and transcribed ad verbatim. Due to a technical error, one interview was not recorded, and the notes taken were used for the analysis. The transcriptions were coded according to recurring topics using thematic analysis. The six steps of the thematic analysis by SH included familiarizing with the data, creating initial codes, exploring themes, reviewing themes, defining and naming these themes and finally producing the report [18]. NVivo 12 software was used for analysis [19]. Secondly, member check of the analysis was performed by MB in line with instructions on peer review of results by the Qualitative Analysis Guide Of Leuven (QUAGOL) [20]. Each participants was invited to provide feedback on their personal interview transcript in general or on specific quotes. Three participants (33%) provided feedback, ranging from none to minor reflections.

## Results

**Fig. 2:**
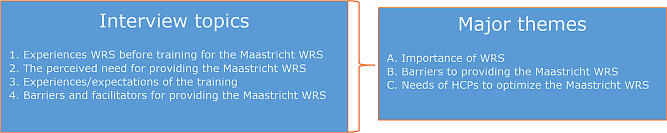
Identified major themes that were derived in the topic-guided interviews

All interviews revealed participants considered WRS generally a valuable part of regular clinical care. Their experiences derived from interviews can be described in three major themes (Fig. 2).

### Importance of WRS

#### Importance of work for patients in general

All participants mentioned the relevance of work for their patients, to feel valuable and to participate in society. Some participants specifically mentioned the difficulties self-employed workers might face in the Netherlands, such as the possibility of not having an occupational physician, and expensive insurances for job loss or work disability.

*I think it is an interesting topic, and I think it is a very important topic.* (P9)

*Work is also a part of satisfaction in your life, you know. When someone feels important, feels valuable, and that someone contributes to society, in fact, how do you call it, self-worth.* (P2)

#### Importance of discussing work

Considering the importance of work for patients, participants mentioned to see the importance to discuss work during consultations in clinical care. However, it was added that topics raised during consultations need to be useful, as any other conversation topic, such as symptoms or medication side effects. One participant even mentioned work still is an underestimated topic.

Some participants had attempted to discuss work during consultations before the Maastricht WRS was introduced, however they had never considered it as part of routine clinical care. During the member check by participants, this was addressed by an additional participant.

Participants also agreed work is still a neglected topic in clinical care, but do understand the importance to discuss work participation as part of clinical care: they previously thought it was sufficient to ask what type of job someone has.

*Yes, because I believe that it [work] is often the ‘neglected child’.* (P1)

*But it is, yeah, it definitely needs being… Yeah, a kind of useful.* (P3)

### Barriers to providing the Maastricht WRS

In interviews, participants mentioned several barriers to providing Maastricht WRS in daily practice. These barriers revoked in the interviews were linked to the determinants of behavioral change, as used in the initial intervention development [13].

#### Attitude

**Lack of knowledge** Untrained participants expressed to have little knowledge about work-related topics and specifically about tailored support. They emphasized the contrast in knowledge they have compared to other professionals, such as occupational physicians.

**Lack of prioritization** Participants mentioned time pressure as a barrier to discuss work in the given consultation time, and therefore they find it difficult to prioritize, especially for patients who recur only (semi) annually. Under these conditions, medical topics are prioritized above work-related topics.

*I think that the biggest barrier for this, is still the time, in a sense of the time you have for a consultation.* (P6)

*But that’s also a bit a challenge […] of course, if someone is sitting across from me, and I have, I have to decide whether I talk about the patient’s pain […] or talk about their work, then I still opt for the first.* (P6)

#### Self-efficacy

**Lack of confidence on how to deal with work-related problems** Participants expressed they feel less competent in discussing work and providing the Maastricht WRS, compared to other professionals. During the member check in the analysis, this was confirmed and participants expressed their doubts about the extent to which they are able and qualified to provide the Maastricht WRS to their patients.

*What I struggle a bit, what I struggle with myself, we are, or rather I am a practitioner on the medical side, the medical nurse side, the curative, the palliative, but you also have HCPs who are on the side of, yes, work. The occupational healthcare.* (P8)

**Lack of information on work status **A few participants indicated the hospital’s electronic patient system provides no standard information about the patient’s work status, even not when patients are newly registered in the hospital. Absence of such information contributes to lack of interest and awareness that work participation should be discussed with patients, especially in medical disciplines where work is not (yet) likely to be addressed. The information absence made participants feel less able to provide the Maastricht WRS.

*Interest… A lack of interest. There is a culture clash between disciplines. It is more difficult for a surgeon. They would be less focused [on work] than those with a non-surgical discipline.* (P8)

#### Social influence

*Patients not taking the initiative *Participants stated work-related topics are usually discussed when patients introduce work-related problems themselves. Some participants mentioned that patients decide which topics are discussed during consultations and work is often not one of these conversation topics. Patients might only address work when their disease is deteriorating and causing more problems at work.

*If you are lucky, someone will say: “I notice in my work that if I have to make this movement [participant showed movement] my shoulder hurts.” Then you examine that shoulder, but then you can quickly ask: “Well, how do you manage this at work?”* (P5)

*Some people say: “It has always been that way [at work], you know?” They do not know better and so do we. Unless the patient comes here and says: “Can I ask you a question?” I say: “Yes, of course, that is why I am here.” And then they bring up a topic of which I think and ask: “Why haven’t you told me this before?”* (P2)

For participants, it appeared important that they are reminded by their own patients as part of the social norm to discuss the topic of work. If patients bring up the topic, the HCP can further discuss work as part of providing the Maastricht WRS.

### Needs of HCPs to optimize the Maastricht WRS

Needs were expressed regarding the three intervention components (see also Fig. 1 for the components).

#### Care pathway

**WRS: an integral part of clinical care **Both trained and untrained participants repeatedly mentioned a need for the Maastricht WRS to become an integral part of regular clinical care, and make work as easy to discuss as other topics. Trained participants who had already the chance to provide the Maastricht WRS in their practice indicated that this care pathway should still be better embedded in clinical care.

*And that we, as a multidisciplinary team, also offer work-related support as a regular part of the management model.* (P2)

*But if our management model [patient-centered care] succeeds, then for us meaning something to people is part of our satisfaction. […] That is why I think it should be a permanent part of our management model, shall we say.* (P2)

**Need for collaboration in the care pathway **The participants also expressed a need to strengthen their collaboration with other HCPs in or outside the hospital in providing the Maastricht WRS to patients, such as the patient’s occupational physician or physiotherapist. Currently, they feel that collaboration with other professionals is lacking. When HCPs refer their patients to other professionals, they are not kept informed about the support the patient receives. This applies not only to referrals within clinical care, but also to referrals outside the hospital, such as to occupational physicians.

*Because I can just say: “You need to go an occupational physician”, but if I thereafter do not know what the occupational physician is initiating, or yes, looks at the problem from another side than how I regard it, then it still has not been useful.* (P4)

*If we as team stand for a patient - so a specialist, the nurses, the physiotherapist, the occupational physician, psychologist or whoever within the multidisciplinary team -, each of them should contribute to get the patient to return to a ‘normal’ state. *(P2)

#### Tools

Some participants who had not yet been trained, wished to receive a set of practical tools to guide them in providing the Maastricht WRS to patients, alluding to tools will make it easier to establish a routine in providing the support. The tools should act as a memory aid during clinical work, where the HCP is free to use these in whichever way desired, to foster discussing work participation eventually becomes a routine. Those trained expressed the developed tools sufficiently meet their needs, but they emphasized that some tools contain current information, making them prone to become outdated, in case legislation or options for support change.

*In the end you should not need to use those cards anymore.* (P3)

*I already have a copy of the conversation cards. I went through them and thought: yes, these are simple, important questions to get a bit of a bit clearer understanding of the situation.* (P2)

#### Training

**Need of relevance to practice **All participants indicated any training should focus (more) on clinical practice. They emphasized the importance of conversation skills, as improved conversation skills could contribute to a better identification of work-related problems. One participant suggested to invite patients or actors to simulate conversations on work-related topics.

*It would be nice to collect real-life problems from practice, or something like that. Because that is in fact a great exercise in order to discuss work with patients. […] Maybe inviting one or two patients who wish to cooperate and have experienced work-related problems.* (P6)

*And training that kind of conversations, you know, I think that is the biggest need. So to speak, role-plays, or watching a video, which is something familiar from recurring patients.* (P3)

Notwithstanding, trained participants appreciated the format of the training as they were stimulated to share personal thoughts and ideas on providing the support to patients, rather than just being instructed by a trainer. They also appreciated that specific work-related situations/needs were discussed in different contexts (e.g. type of employment contract).

**Need for clinical reasoning **Both trained and untrained participants indicated a need to increase their knowledge on work participation and on how to provide the support. For clinicians, this is an essential component to be able to apply clinical reasoning when identifying and addressing needs of patients. Those having not yet participated in the training indicated they were more uncertain about how to elucidate work-related problems and further support their patients in work participation. Specifically they would like to gain insight into sickness legislation in the Netherlands in relation to tailored support to patients, as specific rights and duties differ for paid workers versus self-employed workers. All participants, including those who had followed the training, expressed their need to learn more about the role and responsibilities of occupational physicians for employees.

*I would like to gain more insight to the law and regulations on sickness absence, and what my role is within these regulations.* (P7)

The interviewer’s clarification of HCP’s role in the Maastricht WRS and the possibility to consult another professional in case of severe work participation problems, made participants feel more confident to provide support to patients. For example, in the Maastricht WRS a patient may be referred to a medical specialist in rehabilitation medicine or a social worker. Participants revealed it remained challenging to decide when to refer a person to another professional or when they feel sufficiently comfortable to provide support themselves.

*In case the patient needs expertise from a third party, it is our job to refer the patient to ensure […], so to speak, getting the appropriate support needed.* (P2)

Throughout all interviews and during member check, participants expressed a need to learn more about WRS in general and to reflect on real patient cases. For example, participants deemed it valuable to learn from other stakeholders, such as labor experts, occupational physicians, and insurance physicians.

**Need for reiteration **Finally, to ensure providing the Maastricht WRS to patients will become endurably integrated into clinical care, trained participants mentioned the importance of reiteration and providing support need to become an automatic process. Therefore, training should help establish a routine in providing the support.

*According to me, there are a lot of theories that teach you to repeat, repeat, repeat. Nothing will happen if this remains a one-time request. […] But it also must be trained, and it needs to become an automaticity* (P3)

## Optimizations of the Maastricht WRS intervention

**Fig. 3:**
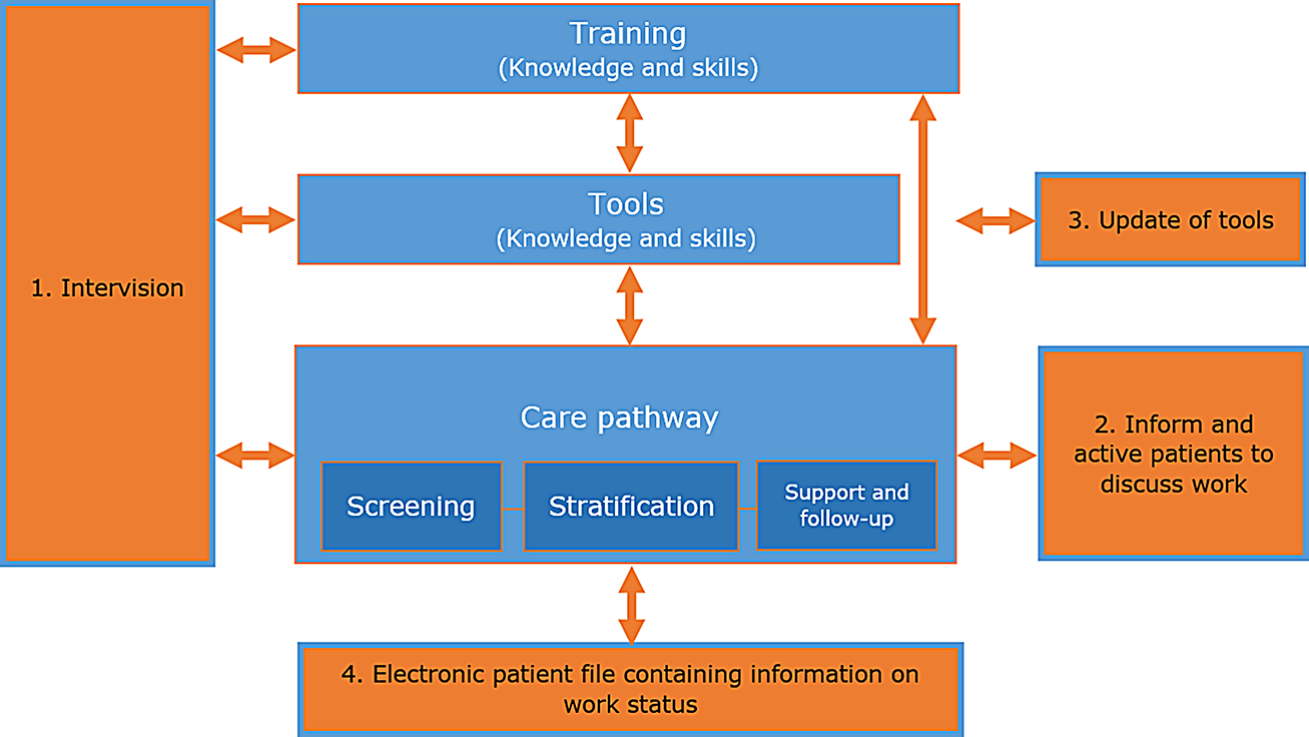
Optimizations added to the initial Maastricht WRS intervention

Action research revealed the Maastricht WRS largely aligns with the experiences of HCPs but also showed that to the original needs assessment (Step 1, IM) of the intervention was not sufficient [13]. Comparing the initial intervention with the experiences of HCPs, a few optimizations are deemed necessary to improve the intervention for its users (Fig. 3).

First, both trained and untrained HCPs expressed their need to increase their knowledge, practice their conversation skills in WRS and to reiterate knowledge and skills. In the initial development, offering ‘intervision sessions’ (peer group reflection meetings, referred to as ‘intervision’) after having been trained, had already been envisioned [13]. In these ‘intervision sessions,’ HCPs learn from peers, share their experiences, and can invite an expert to foster knowledge and skills. Additionally, practical challenges in providing Maastricht WRS in daily practice can be discussed in this setting. However, at the time of this study, ‘intervision’ had not yet been implemented. HCPs expressed a strong need for this, indicating the necessity to facilitate ‘intervision’ for intervention users.

Second, HCPs experience a limited social norm regarding providing systematically WRS as part of usual clinical care. Additionally, they indicated that patients are still only limitedly aware of the possibility to discuss work in the consultation room. To address both issues, an optimization is to better inform patients to discuss work with their HCPs, both through a letter sent home and by information on the screens in the hospital’s waiting room. Once patients are better informed, they can address this topic in the consultation room, contributing to the social norm of HCPs to discuss work-related topics in order to make it common to provide Maastricht WRS as part of regular clinical care.

Third, HCPs expressed satisfaction with the developed tools but emphasized that some tools rely on current information, such as the Map with Options for Work-related Support (e.g., information on legislation, options for referrals) and patient flyer. An optimization is to keep the various tools up-to-date, but this a demanding task. One possibility to reduce demands and thus failures or delays is to create one tool that integrates all information, in addition to the generic conversation tools (which do not contain current information), that is updated if needed.

Fourth and last, HCPs expressed that information on the work status of patients was often lacking, which reduced their self-efficacy to discuss work participation. Another optimization is thus to include information on patient’s work status and work participation in the electronic patient system to promote HCPs self-efficacy.

## Discussion

This study explored how the Maastricht WRS intervention could be optimized. These optimizations were a result of integrating action research principles into Intervention Mapping (IM). More specifically, this study explored experiences of HCPs during early implementation of the intervention that resulted in insights in four additional needs, which can be integrated into Step 1 (Needs assessment) of the IM process of the Maastricht WRS intervention. It is essential that a needs assessment is not perceived as fixed and final, and that intervention planners use a participatory approach not only for Step 1 (IM) but throughout all steps of IM to optimize the intervention. Despite not repeating the entire IM cycle, this approach provided an opportunity to add these optimizations to the intervention while it is in the early implementation phase. This approach was necessary, because, despite formal training, intervention users may still pick up insights about the intervention during the period between development and implementation, leading to the identification of new needs. Additionally, changes in the context may arise, giving input to new needs. In addition, this incremental approach aims to prevent time wastage, costs, or hindrances to the potential benefits for working patients with chronic diseases [21].

In this study, intervention users (HCPs) were involved to share their experiences with the intervention. This participatory action research approach helps intervention planners anticipate real-life problems, barriers, facilitators, and contextual factors [22]. This approach has previously proven suitable for improving healthcare interventions by involving users, such as in improving the quality of primary care by involving deprived communities or enhancing a care pathway for pregnant women with heart disease [23, 24].

The four optimizations identified in this study (1. organizing ‘intervision’ for HCPs, 2. informing and activating patients to discuss work with their HCP, 3. updating initial tools, and 4. including patients’ work status in the electronic patient system) are of practical and logistical nature. It requires creating opportunities within the hospital to facilitate time for ‘intervision sessions’ and making adjustments to the hospital’s systems. Including these optimizations involves both the intervention planners (e.g., updating tools, informing patients) and the hospital management in facilitating (e.g., time and financial resources). However, we are aware that financial resources in healthcare are limited, but it is essential to recognize the potential impact that preventive support to patients for work participation can have, such as improved work productivity and reduced sick leave. Therefore, it is crucial to facilitate HCPs (self-efficacy) to provide the Maastricht WRS to their patients, with an optimized intervention.

Additionally, we are aware of other challenges in healthcare, such as limited time. Hospital management can provide support by sharing its vision on prevention and helping prioritize. Creating a shared goal in which HCPs are involved in healthcare improvements promotes the establishment of a social norm within the organization. In line with motivational theory, this will increase the intention of HCPs to provide the Maastricht WRS to their patients [25].

## Recommendations for further research

Since this study was conducted at only four outpatient clinics in mostly chronic diseases from one university hospital (Maastricht UMC^+^), it is recommended to explore the experiences of a more diverse HCP group. This broader exploration may improve generalizability and could contribute to a better integration of the Maastricht WRS into clinical care. In addition, the Maastricht WRS, including optimizations due to action research, needs to be evaluated in terms of process and effect.

## Strengths and limitations

A first strength is the integration of action research into the (IM) process. This integration allowed the experiences of HCPs to be translated into needs (Step 1, IM), resulting in intervention optimizations. Second, the study sample consisted of several HCPs from different outpatient clinics. An additional strength is the use of QUAGOL for reviewing preliminary results and analyzing data, which contributed to increase the trustworthiness of the findings [20]. Despite not receiving any derogatory input during member check, this indicated no valuable information was lost during interviews and data analysis.

Some limitations should be noted. Firstly, the nine participants may have been more interested in work participation and WRS compared to non-participants, potentially reducing generalizability and external validity (due to selection bias). Secondly, there was variability in the time HCPs could dedicate to the interview; some having slotted an hour for the interview, and others thirty minutes. As these interviews often took place in spare moments during a regular working day, there were interruptions or influences from their working environment, which could affected responses. Lastly, the interview guide was not pilot tested beforehand. However, the interview topic list was based on earlier experiences [13], and provided ample room (tailored to the experience with the intervention) to obtain in-depth insights.

## Conclusions

This study explored how the Maastricht WRS intervention could be optimized. These optimizations were a results of integrating action research into intervention mapping. By exploring experiences of HCPs, it appeared the current Maastricht WRS meets the needs of HCPs, but needs optimization: organizing ‘intervision’ for HCPs, activating the patients to take initiative to discuss their work with their HCP, updating tools for HCPs and including patients’ work status in the electronic patient system. Although integration into regular care is difficult in a context of high workload, this integration is at the same time important for sustainability of the intervention. Thus, action research contributed to improving the provision of these optimizations in the Maastricht WRS intervention to people with chronic diseases.

### Electronic supplementary material

Below is the link to the electronic supplementary material.

**Supplementary Material 1:** Interview guide to collect experiences on providing Maastricht Work-Related Support (WRS) in clinical care

## Data Availability

The data used and analyzed during the current study are available from the corresponding author on reasonable request.

## Abbreviations

HCP: Healthcare professional
Maastricht WRS: Maastricht Work-Related Support intervention
P: Participant
QUAGOL: Qualitative Analysis Guide of Leuven
WRS: Work-related support (in general; not the Maastricht intervention specifically)
