# Supplementary material for: Optimizing the Maastricht Work-Related Support intervention in clinical patient care: the value of integrating action research into intervention mapping
Source: BMC Health Serv Res. 2024 Mar 11;24:325. doi: 10.1186/s12913-024-10752-3 (PMC10929078; doi:10.1186/s12913-024-10752-3)
Supplement: Supplementary file 1 — Supplementary Material 1: Interview guide to collect experiences on providing Maastricht Work-Related Support (WRS) in clinical care [file 12913_2024_10752_MOESM1_ESM.docx]

**Supplementary Table 1.**
*Interview guide to collect experiences on providing Maastricht Work-Related Support (WRS) in clinical care*

| **Topic** | **Open-ended questions** |
| --- | --- |
| Introduction | - Can you please introduce yourself (including your job and medical specialization)? - Can you explain what a normal consultation with patients with a chronic disease looks like? |
| **Topic 1.** experience with WRS before training for the Maastricht WRS | - To what extent did you already provide work-related support before the intervention started? - If so, can you give examples of how you did so? - What do you think is the added value for patients? |
| **Topic 2.**  Perceived need for providing the Maastricht WRS | - Can you explain the extent to which your patients have difficulty with work participation because of their chronic condition? - Can you explain to what extent will your patients appreciate work-related support in clinical care? |
| **Topic 3a.** Experiences of the training session to gain knowledge and skills on providing the Maastricht WRS (only to participants who attended a training session yet) | - What were your expectations prior to your participation to the training sessions? - To what extent were your expectations met? - What did you think of the training sessions? - To what extent have you already applied what you learned? Can you give some examples? - What do you think of these practical tools? - In your opinion, were there any missing elements in the training? (Or what else would you have liked to learn?) Which ones, can you explain? - What elements of the training were unnecessary in your opinion? If so, which ones? |
| **Topic 3b.**  Expectations of the training session to gain knowledge and skills on providing the Maastricht WRS (only to participants who did not attended a training session yet) | - Are there elements, activities or topics you would like to see during training sessions? What would you like to learn? Can you elaborate on these? - Since chronic conditions vary greatly among the outpatient clinics within the hospital, to what extent should the training meet your specific needs regarding the provision of the Maastricht WRS within your clinic? - What information did you receive about the training you will be participating in? - What do you expect from the training prior to your participation? |
| **Topic 4a.** Barriers for providing the Maastricht WRS in practice | - What factors do you think would make it more difficult to pay attention to work? And to provide the Maastricht WRS? - To what extent had these factors been addressed during instruction? If not, how could we do so? |
| **Topic 4b.** Facilitators for providing the Maastricht WRS in practice | - What facilitators do you think would make it easier to pay attention to work? And to provide the Maastricht WRS? |
| Closing comments or questions | - Are there any topics you would like to discuss that have not already been mentioned during this interview? - Would you like to share any other information that you feel is important? - Do you have any other questions? - Would you like to be kept informed about the results of this study? |
